# Uncertainty quantified discovery of chemical reaction systems via Bayesian scientific machine learning

**DOI:** 10.3389/fsysb.2024.1338518

**Published:** 2024-03-08

**Authors:** Emily Nieves, Raj Dandekar, Chris Rackauckas

**Affiliations:** ^1^ Department of Computational Science and Engineering, Massachusetts Institute of Technology, Cambridge, MA, United States; ^2^ Department of Biological Engineering, Massachusetts Institute of Technology, Cambridge, MA, United States

**Keywords:** scientific machine learning, quantitative systems pharmacology, Bayesian machine learning, systems biology, machine learning

## Abstract

The recently proposed Chemical Reaction Neural Network (CRNN) discovers chemical reaction pathways from time resolved species concentration data in a deterministic manner. Since the weights and biases of a CRNN are physically interpretable, the CRNN acts as a digital twin of a classical chemical reaction network. In this study, we employ a Bayesian inference analysis coupled with neural ordinary differential equations (ODEs) on this digital twin to discover chemical reaction pathways in a probabilistic manner. This allows for estimation of the uncertainty surrounding the learned reaction network. To achieve this, we propose an algorithm which combines neural ODEs with a preconditioned stochastic gradient langevin descent (pSGLD) Bayesian framework, and ultimately performs posterior sampling on the neural network weights. We demonstrate the successful implementation of this algorithm on several reaction systems by not only recovering the chemical reaction pathways but also estimating the uncertainty in our predictions. We compare the results of the pSGLD with that of the standard SGLD and show that this optimizer more efficiently and accurately estimates the posterior of the reaction network parameters. Additionally, we demonstrate how the embedding of scientific knowledge improves extrapolation accuracy by comparing results to purely data-driven machine learning methods. Together, this provides a new framework for robust, autonomous Bayesian inference on unknown or complex chemical and biological reaction systems.

## 1 Introduction

Mechanistic models in Quantitative Systems Pharmacology (QSP) offer more interpretability than purely data-driven machine learning approaches, but practitioners often lack complete knowledge of the underlying systems. Methods of Scientific Machine Learning (SciML) account for this epistemic uncertainty by mixing neural network techniques with mechanistic modeling forms to allow for the automated discovery of missing components in mechanistic models Rackauckas et al. (2021); Dandekar et al. (2020b). In addition to interpretability, SciML models also offer superior prediction extrapolation beyond the domain in which they were trained, making them attractive candidates for QSP and systems biology applications.

The Chemical Reaction Neural Network (CRNN) is a recently proposed SciML method aimed at discovering chemical reaction pathways from concentration time course data without prior knowledge of the underlying system Ji and Deng (2021). Specifically, the CRNN is a Neural ODE (Ordinary Differential Equation) architecture in that the neural network learns and represents the system of ODEs that comprise the reaction network Chen et al. (2018). The CRNN is a neural network architecture which is designed to be a flexible representation of mass action kinetics models, thus imposing prior known constraints about the guiding equations of chemical reaction models while allowing for interpretable weights which represent the individual reaction weights. The weights of the embedded neural networks represent the stoichiometry of the chemical species and kinetic parameters of the reactions. Optimization of the neural network weights thus leads to the discovery of the reaction pathways involved in the network. As QSP models often involve complex, interconnected reaction networks such as signaling or metabolic reactions, the CRNN may aid in the development of these models when the reactions are unknown or incompletely known.

The learned reaction system and the kinetic parameters of the CRNN are obtained in a deterministic manner, however to build trust in predictions and help experts understand when more training data is needed, the uncertainty of the predictions should also be quantified. For this purpose, Bayesian inference frameworks can be integrated with the CRNN. Bayesian methods obtain estimates of the posterior probability density function of neural network parameters allowing for an understanding of prediction uncertainty. It is the goal of this work to extend the CRNN with a Bayesian framework to increase its utility in QSP and other scientific domains. This is similar to the work by Li et al. in which they also extend the CRNN to include a Bayesian framework Li et al. (2023), but here we use a different methodology for sampling the posterior.

Recently, there has been an emergence of efficient Bayesian inference methods suitable for high-dimensional parameter systems, specifically methods like the No U-Turn Sampler (NUTS) Hoffman and Gelman (2014), Stochastic Gradient Langevin Descent (SGLD) Welling and Teh (2011) and Stochastic Gradient Hamiltonian Monte Carlo (SGHMC) Chen et al. (2014). These methods are based on Markov Chain Monte Carlo (MCMC) sampling and utilize gradient information to obtain estimates of the posterior. A number of studies have explored the use of these Bayesian methods to infer parameters of systems defined by ODEs Lunn et al. (2002); von Toussaint (2011); Girolami (2008); Huang et al. (2020), while others have used Bayesian methods to infer parameters of neural network models Jospin et al. (2020); Maddox et al. (2019); Izmailov et al. (2019).

Specifically for Neural ODEs, the addition of Bayesian methods presents technical complexity as the training of the neural network is also tied to the solving of the ODE system. However recent work has demonstrated the feasibility of this approach Dandekar et al. (2020a). To apply a Bayesian framework to a Neural ODE model, the authors make use of the Julia programming language which allows differential equation solvers Rackauckas et al. (2020); Rackauckas and Nie (2017) to be combined with Julia’s probabilistic programming ecosystem Ge et al. (2018); Xu et al. (2019). In this study, we similarly leverage the differential and probabilistic programming ecosystem of the Julia programming language to integrate the CRNN with Bayesian inference frameworks.

While powerful in their ability to estimate the posterior distribution, Bayesian inference methods may suffer from inefficient sampling of the posterior. The inefficiency often stems from the pathological curvature of the parameter space that leads to “bouncing” around minima. To address this, a new optimizer was proposed by Li et al. that combines an adaptive preconditioner with the SGLD algorithm Li et al. (2015). The preconditioner performs a local transform of the parameter space such that the rate of curvature is the same in all directions, allowing for smoother, faster descent.

In this study, we propose combining the CRNN with the more efficient preconditioned SGLD optimizer Li et al. (2015) to discover chemical reaction networks and quantify the uncertainty of the learned reaction parameters, allowing for its more robust use in QSP and other scientific domains. We apply this Bayesian SciML method to a systems biology pathway to demonstrate its application to QSP. We also compare the results to those generated using purely data-driven machine learning methods to further demonstrate the advantages of SciML methods.

## 2 Methods

Similarly to the originally proposed CRNN Ji and Deng (2021), we define the Chemical Reaction Neural Network (CRNN) to represent the following elementary reaction:

$$\nu_{A}A+\nu_{B}B\to \nu_{C}C+\nu_{D}D$$ (1)

where *ν* refers to the stoichometric coefficients of the respective chemical species.

The law of mass action applied to the example reaction prescribed in Equation (1) leads to the following expression for the reaction rate *r*:

$$r=exp\left(lnk+\nu_{A}\;ln\left[A\right]+\nu_{B}\;ln\left[B\right]+0\;ln\left[C\right]+0\;ln\left[D\right]\right)$$ (2)

where k is the rate constant of the reaction and [A] refers to the concentration of chemical species A.

**FIGURE 1:**
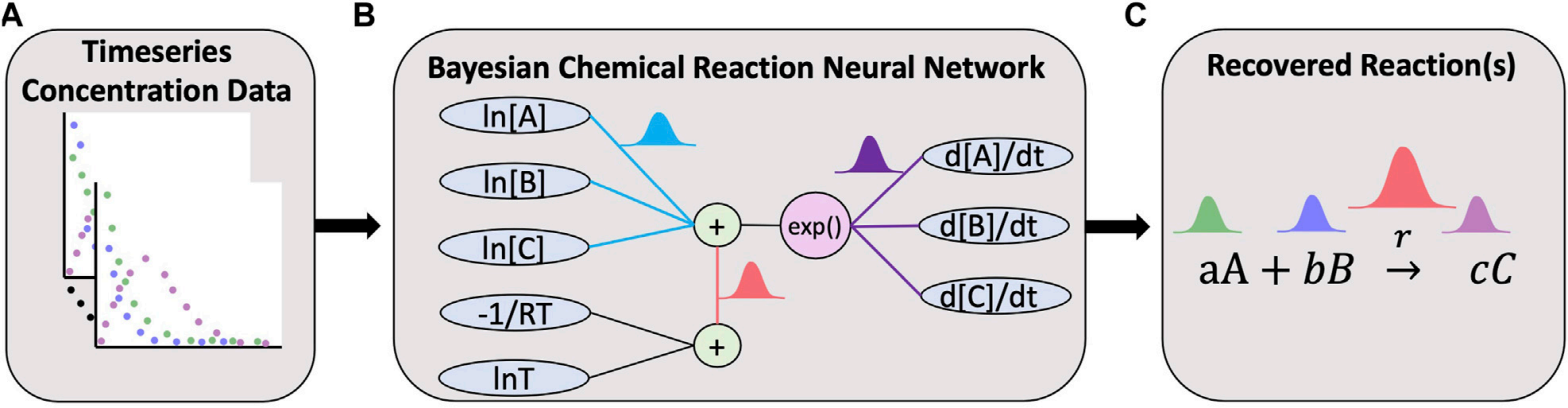
Overview of the Bayesian chemical reaction network which uses timeseries concentration data **(A)** to train a constrained neural network **(B)** that uses a preconditioned SGLD optimizer to reconstruct the reaction network and estimate the uncertainty in the learned stoichiometry and reaction rates **(C)**.

This elementary reaction can be represented by a neuron governed by *y* = *σ*(*wx* + *b*) where *w* are the weights, *y* is the neuron output, *x* is the input to the neuron and *σ* is the activation function. The inputs are the concentration of the species in logarithmic scale, and the output is the production rate of all species: $\left[\dot{A},\dot{B},\dot{C},\dot{D}\right]$ . The input layer weights denote the reaction orders, i.e., [*ν*_*A*_, *ν*_*B*_, 0, 0] for the reactants [*A*, *B*, *C*, *D*] respectively. The output layer weights denote the stochiometric coefficients, i.e., [−*ν*_*A*_, − *ν*_*B*_, *ν*_*C*_, *ν*_*D*_] for [*A*, *B*, *C*, *D*] respectively. The bias denotes the rate constant in the logarithmic scale. This is depicted in Figure 1.

A reaction network with multiple chemical reactions can be denoted by a CRNN with a hidden layer. Since the weights and biases of a CRNN are physically interpretable, it is a digital twin of a classical chemical reaction neural network. In this study, we employ Bayesian inference analysis on this digital twin to discover chemical reaction pathways in a probabilistic manner.

Considering the vector of chemical species *Y* to be varying in time, we aim to recover a CRNN which satisfies the following equation:

$$\dot{Y}=CRNN\left(Y\right)$$ (3)

where $\dot{Y}$ and is the rate of change of the concentration of chemical species Y.

Thus, by representing the ODE governing the variation of the chemical species *Y* by a neural network, we can solve Equation (3) using powerful, purpose built ODE solvers provided by the differential programming ecosystem of the Julia programming language Rackauckas and Nie (2017). The solution we obtain by solving the ODE is denoted by *Y*_CRNN_(*t*). In this study, the loss function between the actual species concentrations and the predicted values is governed by the Mean Absolute Error (MAE) with L2 regularization, and given by

$$L\left(\theta_{t}\right)=MAE\left(Y_{\text{CRNN}}\left(t\right),Y_{\text{data}}\left(t\right)\right)+\lambda_{L2}\left(\theta_{t}\cdot \theta_{t}\right)$$ (4)

where *θ*_*t*_ are the weights of the CRNN at time *t*, *λ*_*L*2_ is the L2 regularization coefficient, *Y*_*CRNN*_(*t*) are the CRNN predicted values of the species concentrations at time t, and *Y*_*data*_(*t*) is the true species concentration at time t.

To incorporate uncertainty into our predictions, we augment the gradient descent algorithms with a Bayesian framework. Our algorithm is shown in Algorithm 1. We use a variation of the Stochastic Gradient Langevin Descent (SGLD) algorithm Welling and Teh (2011) and add a preconditioning term *G*(*θ*_*t*_), which adapts to the local geometry leading to more efficient training of deep neural networks as noted in Li et al. (2015). In Algorithm 1, *β* is a smoothing factor, *λ* is a small constant that may be tuned, n is the number of training samples, and ⊙ and ⊘ refer to element-wise vector product and division respectively.

Similarly to Li et al. (2015), we utilize a decreasing step-size that’s given by the following equation:

$$\epsilon \left(t\right)=\alpha +{\left(b+t\right)}^{-\gamma }$$ (5)

where *ϵ*(*t*) is the step-size, t is the epoch number, and *α*, b and *γ* are tunable parameters.

Algorithm 1 Preconditioned SGLD applied to Neural ODE.

**Inputs:** *ϵ*_*t*_(*t* = 1: *T*), *λ*, *β*, *λ*_*L*2_ **. Outputs:** *θ*_*t*_. **Initialize:** *V*_0_ = 0, *θ*_*t*_: Xavier NN initialization. **for t in 1: T do**
sample batch with size *n*
*Y*_CRNN_(*t*) = ODESolve(*CRNN*(*Y*, *θ*_*t*_), *Y*_0_)
*L*(*θ*_*t*_) = MAE(*Y*_CRNN_(*t*), *Y*_data_(*t*)) + *λ*_*L*2_(*θ*_*t*_ ⋅*θ*_*t*_)

$V\left(\theta_{t}\right)=\left(1-\beta \right)V\left(\theta_{t-1}\right)+\beta \nabla L_{\theta_{t}}⊙\nabla L_{\theta_{t}}$

$G\left(\theta_{t}\right)=diag\left(1⊘\left(\lambda I+\sqrt{V(\theta_{t}})\right)\right)$

$\theta_{t+1}=\theta_{t}-\epsilon_{t}\left(\nabla L_{\theta_{t}}G\left(\theta_{t}\right))+\frac{1}{\text{n}}N(0,\epsilon_{t}G\left(\theta_{t}\right)\right)$

**end for**

In this algorithm, the induced noise and step-size decay to zero as the training proceeds. By adding Gaussian noise to each step of the gradient descent, the optimizer finds a local model, but never converges due to the induced noise. The decayed learning rate also prevents the optimizer from leaving the model, leading to random walking around the mode. The process after settling in a local model is the sampling phase, and in this phase we draw parameter posterior samples. The point at which the optimizer enters the sampling phase can be determined by observing when the loss function stagnates. Thus, the parameters *θ* of the CRNN obtained during the sampling phase lead to the parameter posterior which ultimately helps in encoding uncertainty in the prediction of the chemical reaction pathways.

## 3 Results

### 3.1 Case 1: simple reaction network

We first consider the chemical reaction given in Searson et al. (2014) comprising of five species: [*A*, *B*, *C*, *D*, *E*] involved in four reactions, described in Table 1. Similar to Ji and Deng (2021), a total of 100 synthetic datasets were simulated. Initial conditions of the first and second species, A and B, were randomly sampled from 0.2–1.2. Initial conditions for species C, D, and E were set to 0. Each dataset is comprised of 100 time points. Out of the 100 datasets, 90 are used for training and the remaining are reserved for validation. Further, mini-batching is employed to accelerate the training process and add additional regularization. As the validation loss function stagnates, early stopping is employed. Mini-batching and early stopping prevent the CRNN from overfitting the training data. Hyperparameters were tuned via manual searching to find values that minimized error and improved convergence. Here, we choose *λ* = 1*e* − 6, *β* = 0.9, and *λ*_*L*2_ = 1*e* − 5. For the step-size parameters we choose *α* = 0.001, *b* = 0.15, and *γ* = 0.005.

**TABLE 1:** Case 1 ground truth reactions.

Equation	Rate
2 *A* → *B*	0.1
*A* → *C*	0.2
*C* → *D*	0.13
*B*+ *D* → *E*	0.3

**FIGURE 2:**
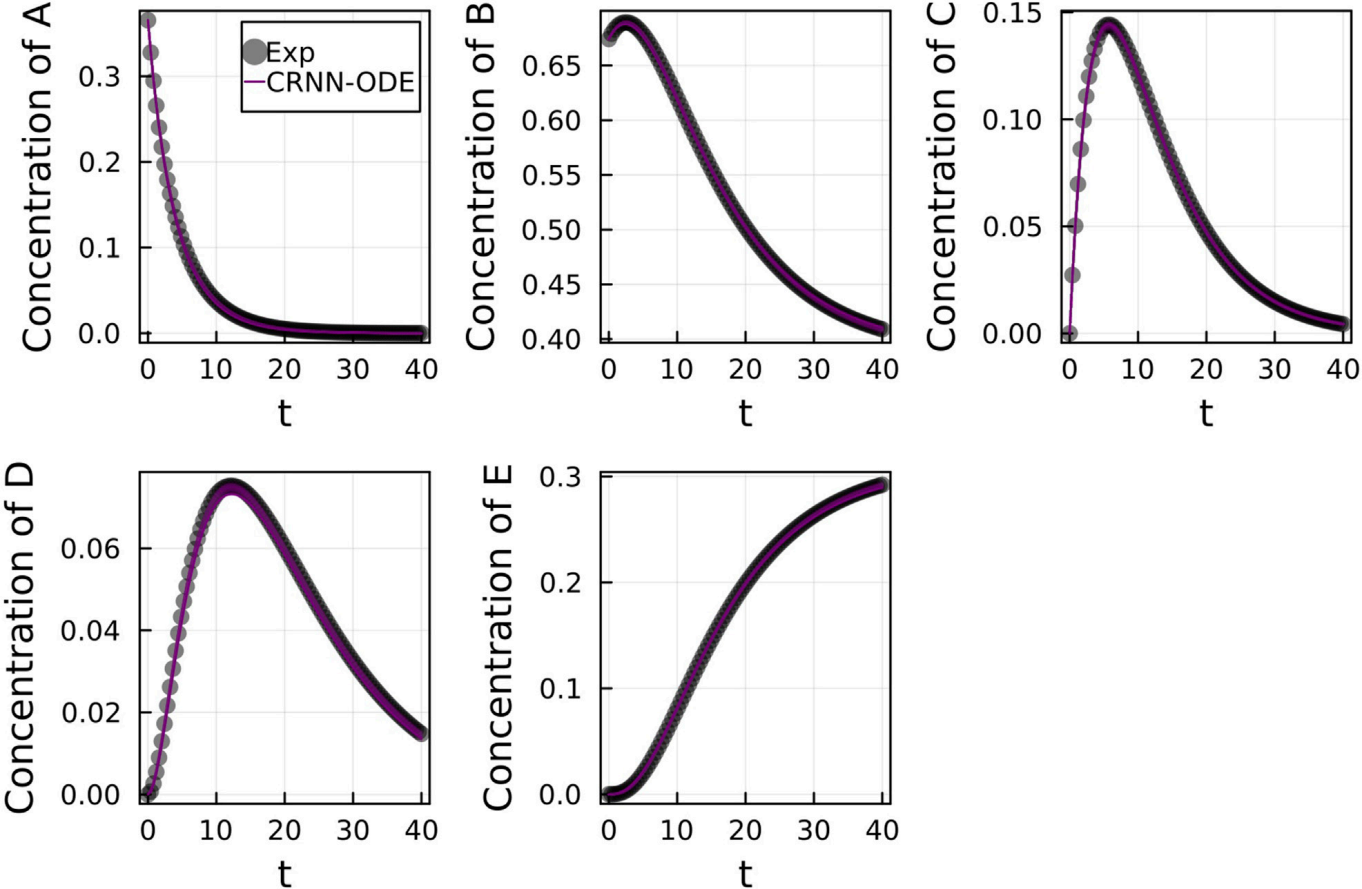
Comparison of the Bayesian chemical reaction neural network prediction to the data for the species **(A–E)** in the four reactions described in Case 1. No noise was added to the data. A total of 500 posterior sample predictions are superimposed on the data.

Figure 2 shows the comparison of the Bayesian Neural ODE prediction compared to the data for the species A, B, C, D, and E in the four reaction system described in Case 1. A total of 500 posterior sample prediction are superimposed on the data; and a good agreement is seen between all trajectories and the data.

**FIGURE 3:**
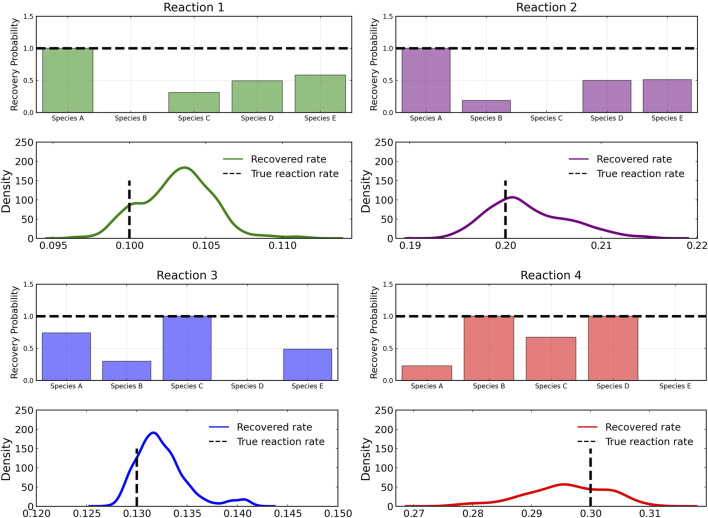
Reactant recovery probability of chemical species (top) and posterior distributions of learned reaction rates (bottom) for the four reactions described in Case 1. No noise was added to the training data and a posterior set of 1,000 samples was chosen for the estimation.

Figure 3 shows the recovery probability of species A, B, C, D, and E in the four reactions described in Case 1, obtained using the preconditioned SGLD described in Algorithm 1. A posterior set of 1,000 samples was chosen for the estimation. A species is considered to be present in a particular reaction if its weight is greater than 1*e* − 4. The probability a species is contributing to a reaction is given by the ratio of the number of samples in which the species is present in that reaction to the total number of posterior samples (1,000 in this case).

However, even if the probability of a species to be present in a reaction is high, its weight can be low compared to other species. Thus, we define a score metric for a species *i* in reaction *j* governed by weight *w*_*ij*_ to be as follows:

$${score}_{ij}=p_{ij}\left(\sum w_{ij}/maximum\left(\sum w_{:j}\right)\right)$$ (6)

where *p*_*ij*_ is the recovery probability for the species shown in Figure 3, *∑w*_*ij*_ is the summation of the weight values of species *i* for reaction *j* over all posterior samples and maximum(*w*_:*j*_) is the maximum value of this summation for the reaction *j* among all species.

**FIGURE 4:**
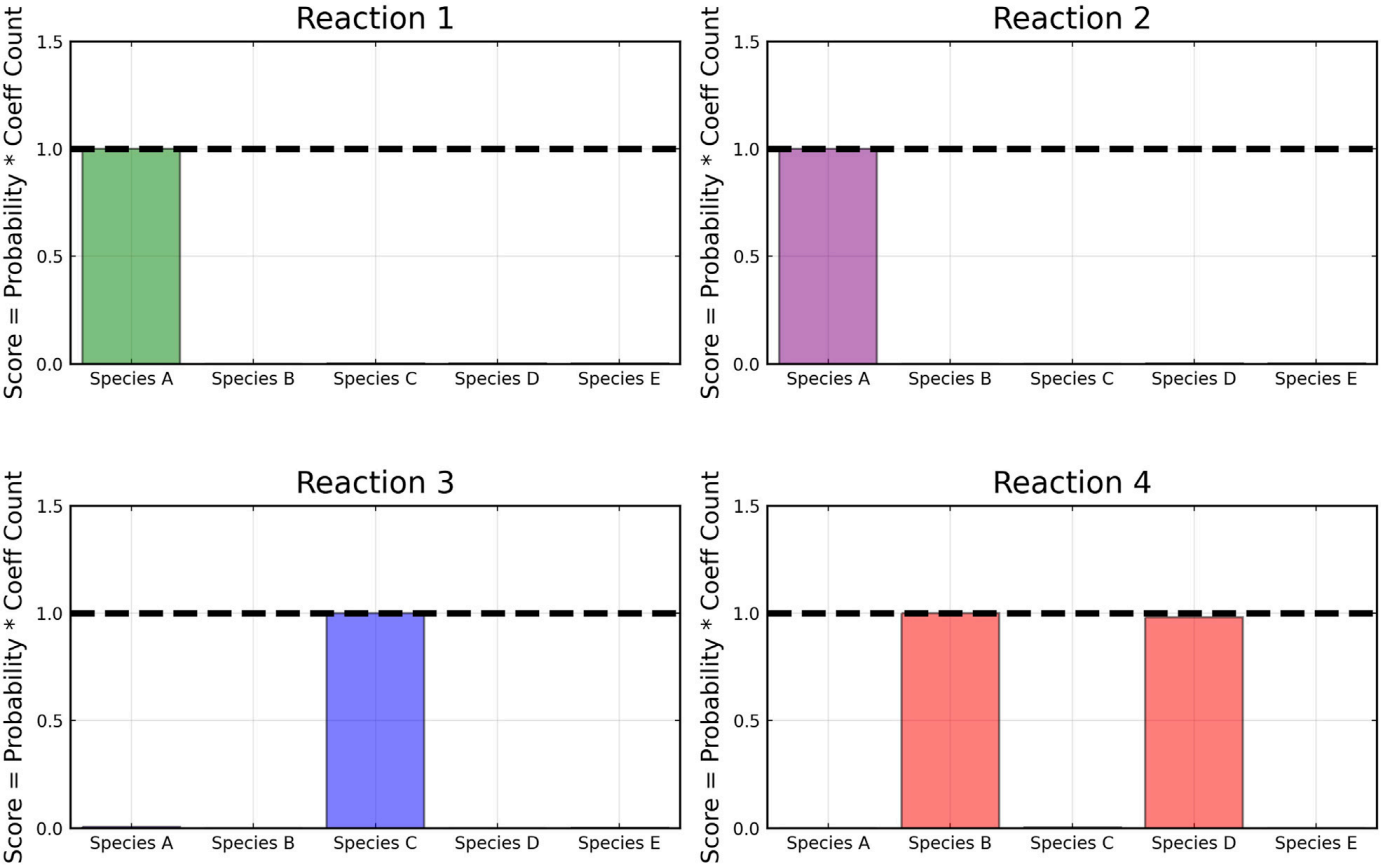
Recovered score metric for each of the four reactions shown in case 1. Score metrics are calculated using Equation 6 and represent the weighted probability that a species is a reactant of each reaction. No noise was added to the data.

From the recovered score metrics shown in Figure 4, we can see that we obtain a sparser discovery of the reaction pathways; which match with the data shown in Table 1.

#### 3.1.1 Moderately noisy data

**FIGURE 5:**
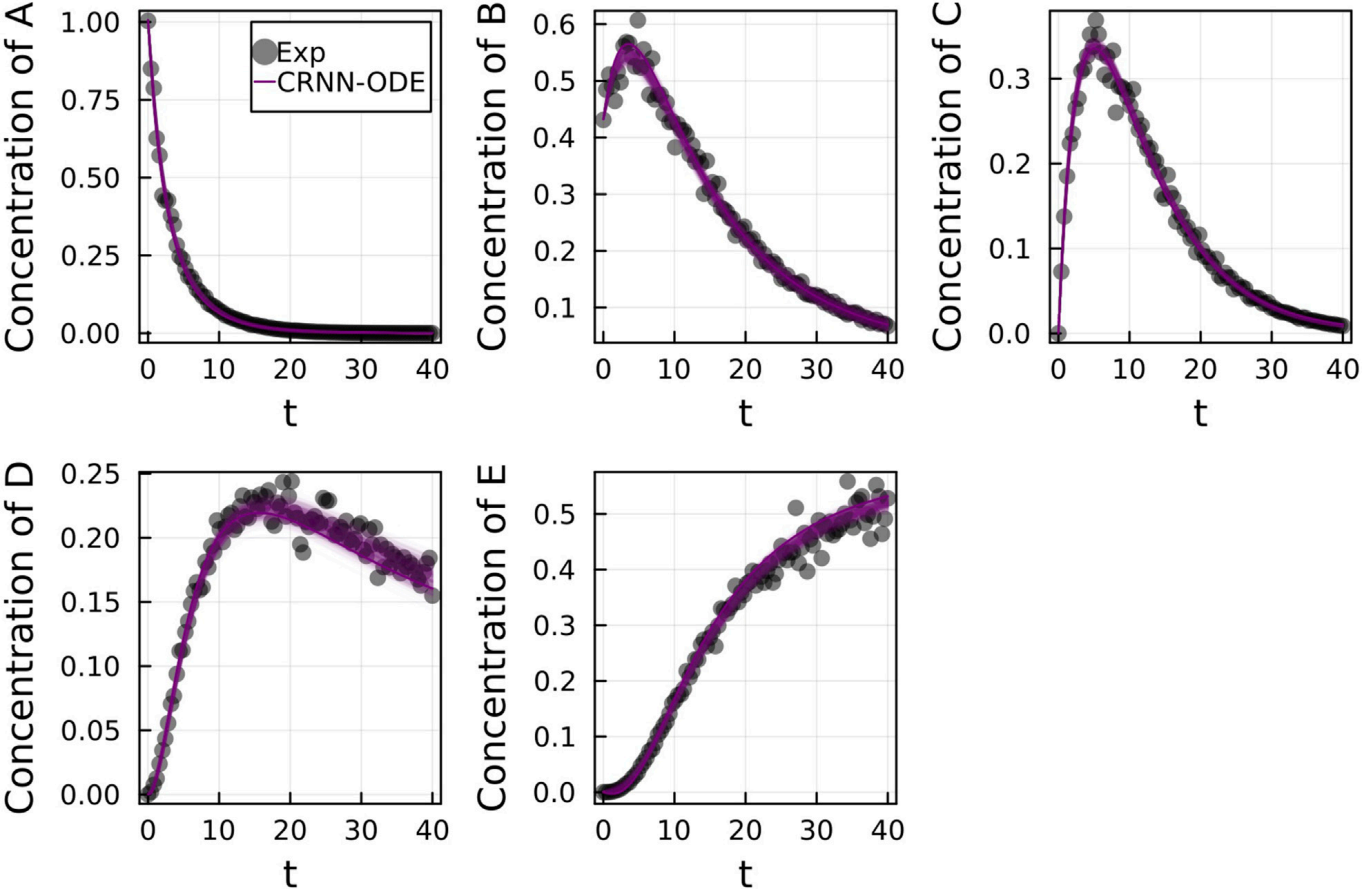
Comparison of the Bayesian chemical reaction neural network prediction to the data for the species **(A–E)** in the four reactions described in Case 1. The model was trained with moderately noisy data, where the standard deviation of the noise was set to 5% of the concentrations. A total of 500 posterior sample predictions are superimposed on the data.

We subsequently train on moderately noisy data with standard deviation of the noise being 5% of the concentrations. The data is visualized in Figure 5. Figure 5 shows the comparison of the Bayesian Neural ODE predictions to the data for 500 posterior samples. Even with the addition of noise, our methodology can correctly capture the time course of all the chemical species.

**FIGURE 6:**
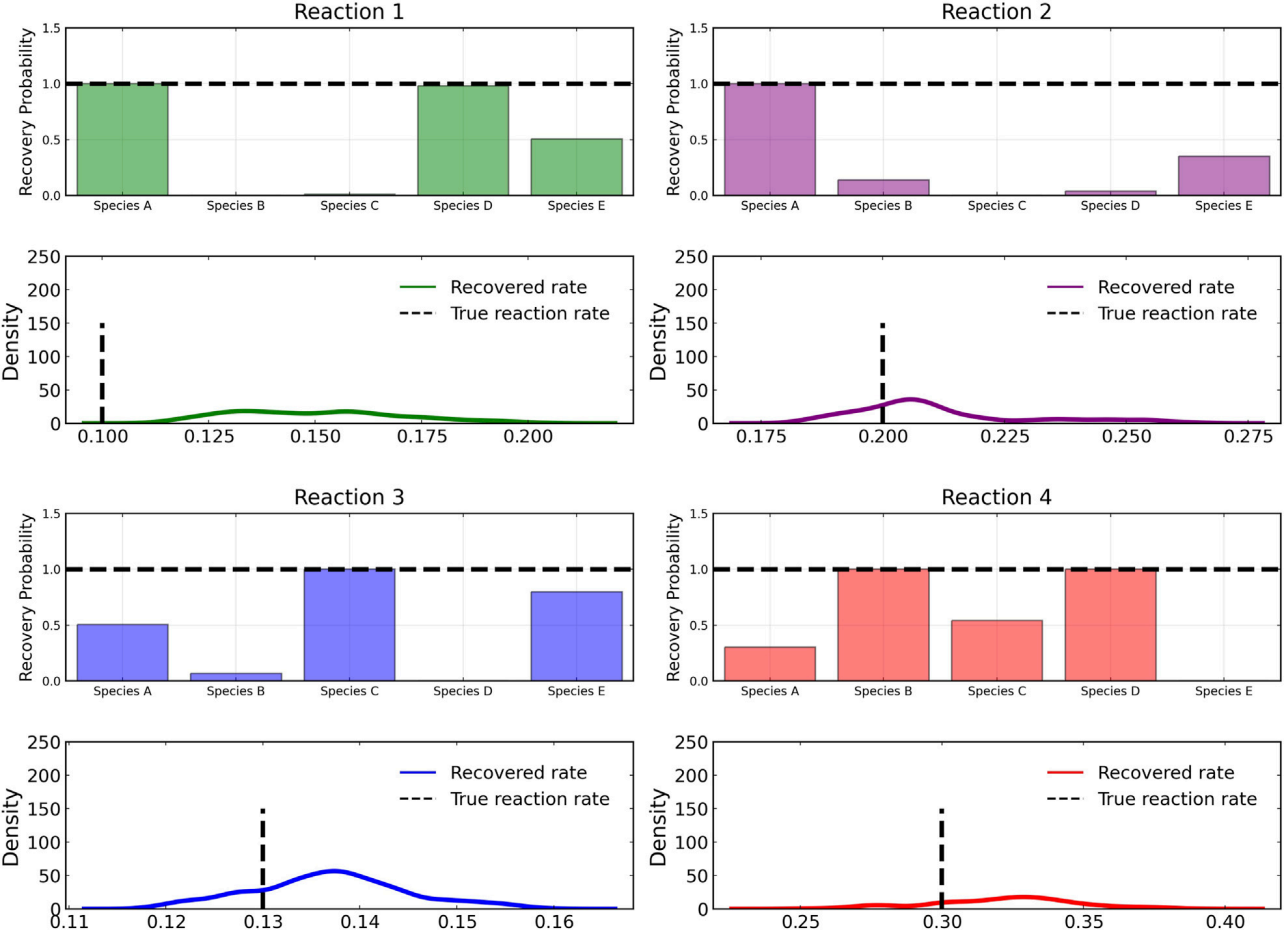
Reactant recovery probability of chemical species (top) and posterior distributions of learned reaction rates (bottom) for the four reactions described in Case 1. The model was trained with moderately noisy data, where the standard deviation of the noise was set to 5% of the concentrations. A posterior set of 1,000 samples was chosen for the estimation.

**FIGURE 7:**
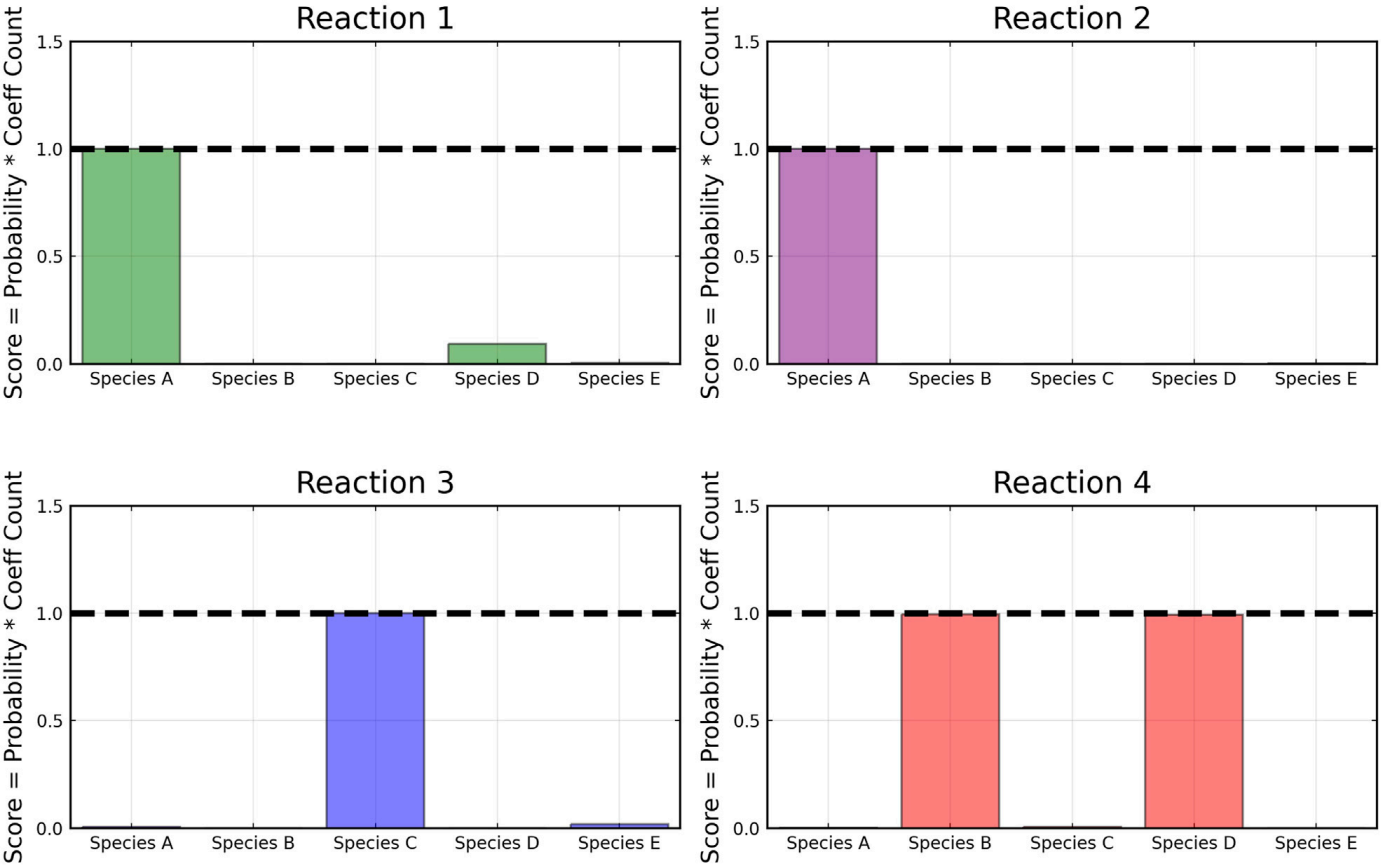
Recovered score metrics for each of the four reactions shown in case 1. Score metrics are calculated using equation 6 and represent the weighted probability that a species is a reactant of each reaction. The model was trained with moderately noisy data, where the standard deviation of the noise is set to 5% of the concentrations.

The reactant recovery probability and the score metric plots are shown in Figure 6, Figure 7 respectively. Even with the presence of noise, the probability plots are seen to assign higher probability to the correct reaction pathways. The score plots accurately predict the reaction pathways as seen in the data (Table 1). Figure 6 also shows the posterior distribution of the reaction rates. Even with the addition of the noise, the distributions are centered close to the true reaction rate for all reactions except reaction 1, which still contains the true reaction rate within its distribution.

#### 3.1.2 Highly noisy data

To test the limits of the Bayesian Neural ODE framework, we subsequently train on highly noisy data with the standard deviation of the added noise being 50% of the concentrations. The Bayesian Neural ODE predictions are visualized in Supplementary Figure S1 alongside the data. We observe that even with 50% noise, our methodology captures the time course data well.

The reactant recovery probability and the score metric plots are shown in Supplementary Figures S2, S3 respectively. Even with the addition of 50% noise, the Bayesian CRNN still accurately predicts the reaction pathways. Similarly, the posterior distributions of the reaction rates shown in Supplementary Figure S2 contain the true reaction rates, however they are no longer centered at the true reaction rate, with the exception of reaction 4.

#### 3.1.3 Comparison to SGLD

We compare our results to the standard SGLD optimizer to demonstrate the effect of the preconditioner. Supplementary Figure S4 shows training and validation loss over epochs for both the pSGLD and the standard SGLD. The preconditioned algorithm allows for faster entry into the sampling phase, entering around 6,500 epochs before the SGLD.

**FIGURE 8:**
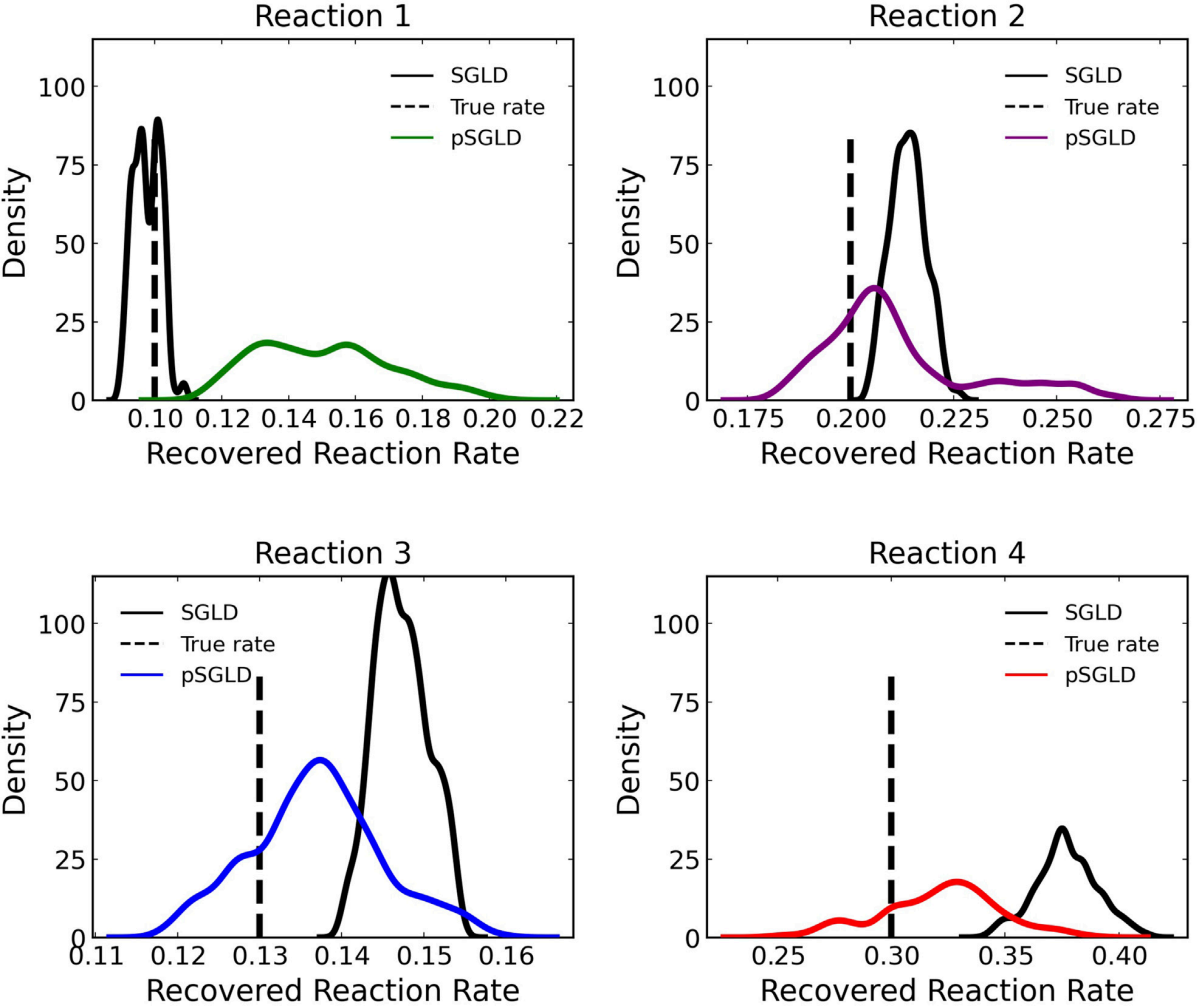
Comparison of estimated posterior of recovered reaction rates between the preconditioned SGLD and SGLD optimizer for the four reactions in case 1 where noise with a standard deviation of 5% was added.

Figure 8 compares the learned reaction rates in the case where 5% noise is added to the training data between the pSGLD and the SGLD. From this figure we can see that in general the pSGLD achieved a posterior that more closely matched the true reaction rates. The average percent deviation of the samples from the true reaction rates for the pSGLD were 50.49% ± 20.01, 7.44% ± 7.73, 6.70% ± 4.68, and 9.50% ± 6.14 for reactions 1, 2, 3, and 4 respectively. The average percent deviation from the true reaction rates for the standard SGLD were 3.78% ± 2.60, 6.96% ± 2.17, 13.27% ± 2.49, and 25.65% ± 4.52 for reactions 1, 2, 3, and 4 respectively. The pSGLD is similar or better in all reactions except for reaction 1. In reaction 1, the pSGLD has lower confidence, as shown in the low density, wide coverage of the posterior samples. In the other reactions, the SGLD demonstrates higher confidence in incorrect reaction rates whereas the pSGLD better represents the uncertainty by estimating a posterior that is centered at the true reaction rate.

#### 3.1.4 Comparison to LSTM

We also compared the Bayesian CRNN to a purely data-driven machine learning approach, in this case a Long Short-Term Memory (LSTM) model. This architecture predicts a sequence, in this case the timecourses for the chemical reaction species. We utilize the LSTM modules from Flux.jl Innes (2018), and connect two LSTM modules with 200 hidden nodes each to a densely connected linear output layer. Similarly to the neural ODE, we train for 2000 epochs with the ADAM optimizer (learning rate tuned to 0.001).

**FIGURE 9:**
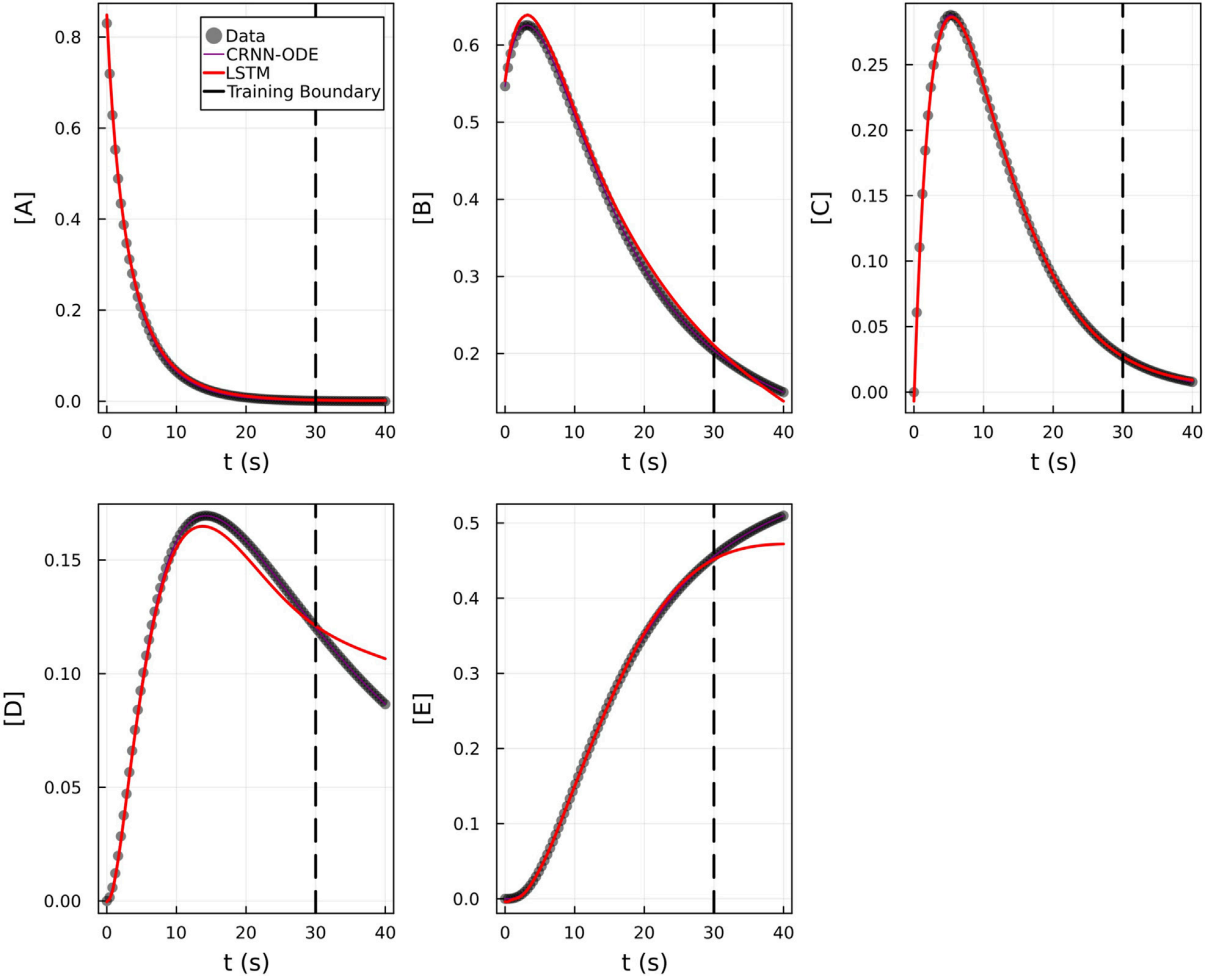
Comparison of LSTM model with Bayesian CRNN (chemical reaction neural network) for chemical species **(A–E)**. Only time points 0–30 were used for training as depicted by the training boundary. Time points 30–40 were used for comparison of the accuracy of extrapolation beyond training time points.

From Figure 9, we can see that the results of the CRNN and LSTM model are nearly indistinguishable in the training region, but the LSTM model diverges from the true concentration values in the extrapolation region while the CRNN matches the true values. The mean absolute percent error in the extrapolation region was 9.0% ± 3.2 for the CRNN in comparison to 90.93% ± 58.83 for the LSTM-based model.

#### 3.1.5 Comparison to neural ODE

Additionally, we compared the Bayesian CRNN to a purely data-driven neural ODE approach, where the structure does not include embedded scientific knowledge. For the neural ODE, we built a densely connected neural network with two hidden layers with 50 nodes each and hyperbolic tangent activation functions. The neural network was trained for 2000 epochs with the ADAM optimizer (learning rate set to 0.001). Here we compare the ability of the learned networks to extrapolate beyond the time points used for training. We use timepoints 0–30s for training and then use the trained system to predict concentrations 30–40 extrapolate to timepoint 40.

The results are summarized in Supplementary Figure S5 and demonstrate the superior ability of the CRNN to accurately predict beyond region used for training. The mean absolute percent error in the extrapolation region was 9.0% ± 3.2 for the CRNN in comparison to 254.7% ± 109.8 for the neural ODE.

### 3.2 Case 2: EGFR- STAT3 pathway

To further test the capabilities of the Bayesian CRNN, we attempted to recover the reaction network governing the EGFR- STAT3 signaling pathway Bidkhori et al. (2012). Our simplified representation of this pathway consists of seven chemical species and six reactions listed in Table 2. Here we omit the reverse reactions because they cannot be identified from the forward reactions with this framework.

**TABLE 2:** Case 2 EGFR-STAT3 reactions.

Equation	Rate
*EGF* − *EGFR* + *EGF* − *EGFR* → *EGF* − *EGFR*2	10*uM* ^−^1*s* ^−^1
*EGF* − *EGFR*2 → *pEGF* − *EGFR*2	2.014 s^−^1
*pEGF* − *EGFR*2 + *STAT*3 → *pEGF* − *EGFR*2 − *STAT*3	5.5*uM* ^−^1*s* ^−^1
*pEGF* − *EGFR*2 − *STAT*3 → *pEGF* − *EGFR*2 + *STAT*3	11.74 s^−^1
*pEGF* − *EGFR*2 − *STAT*3 → *pEGF* − *EGFR*2 + *pSTAT*3	0.4 s^−^1
*pSTAT*3 + *pSTAT*3 → *pSTAT*3 − *pSTAT*3	20*uM* ^−^1*s* ^−^1

In this case, unlike the simple system in case one, the reaction rates and concentrations vary by one or two orders of magnitude. To account for this difference in scale, we adjust our loss function to use mean absolute percent error (MAPE) instead of MAE. Additionally to allow for more reactions and chemical species, we use L1 regularization on the weights that correspond to the stoichiometric coefficients of the reactions *θ*_*v*_, as these would comprise a sparse matrix as the reaction network grows. We continue to use L2 regularization for the weights that correspond to the reaction rates, *θ*_*r*_. This change to the loss function is depicted in the equation below.

$$L\left(\theta_{t}\right)=MAPE\left(Y_{\text{CRNN}}\left(t\right),Y_{\text{data}}\left(t\right)\right)+\lambda_{L1}\left(\sum |\theta_{v}|\right)+\lambda_{L2}\left(\theta_{r}\cdot \theta_{r}\right)$$ (7)

We sample from the initial conditions ranging from 0 to 1 to obtain 300 training examples. With 5% Gaussian noise added to simulated training data. For training, we set *λ* = 1*e* − 8, *β* = 0.9, the L1 coefficient (*λ*_*L*1_) = 1e-4 and the L2 coefficient (*λ*_*L*2_) = 1e-5. Step-size hyperparameters *α*, b, and *γ* were set to 0.001, 0.15, and 0.005 respectively.

**FIGURE 10:**
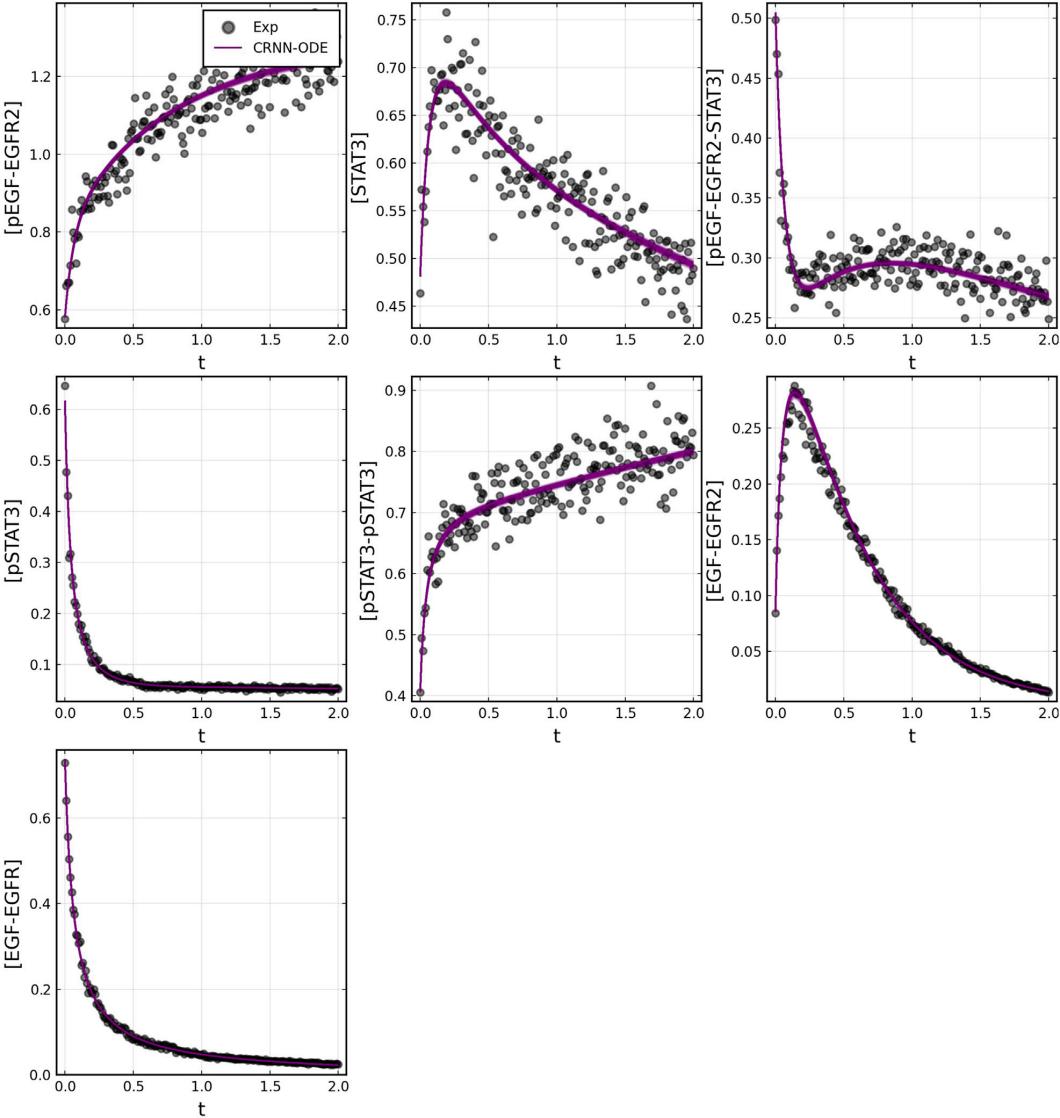
Comparison of Bayesian chemical reaction neural network prediction compared to training data for EGFR-STAT3 pathway described in case 2. Gaussian noise with standard deviation of 5% was added to the training data. 500 posterior sample predictions are superimposed on the data.

**FIGURE 11:**
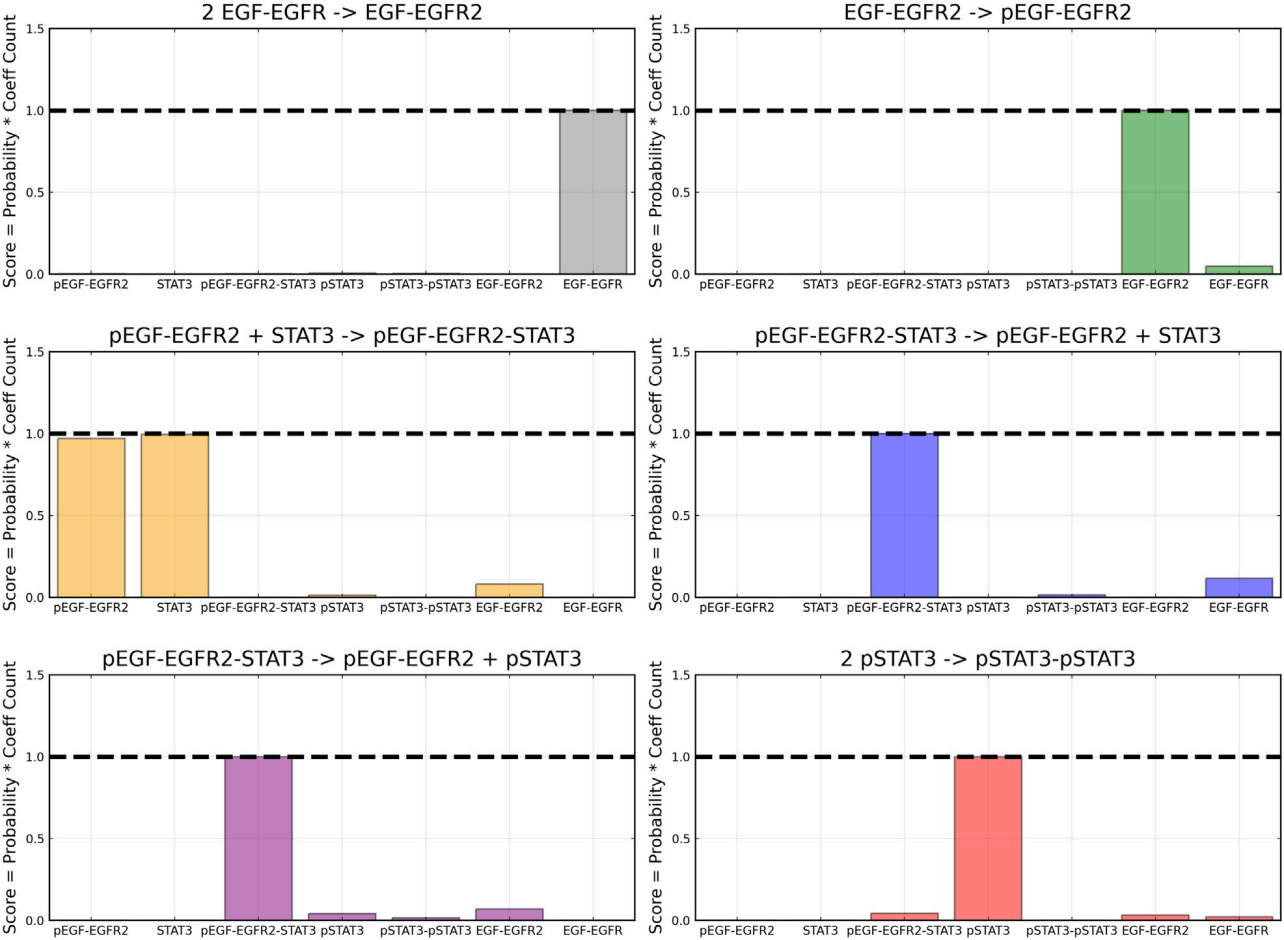
Recovered score metric for each of the six reactions included in case 2. Score metrics are calculated using equation 6 and represent the weighted probability that a species is a reactant of each reaction. Gaussian noise with standard deviation of 5% was added to the training data.

**FIGURE 12:**
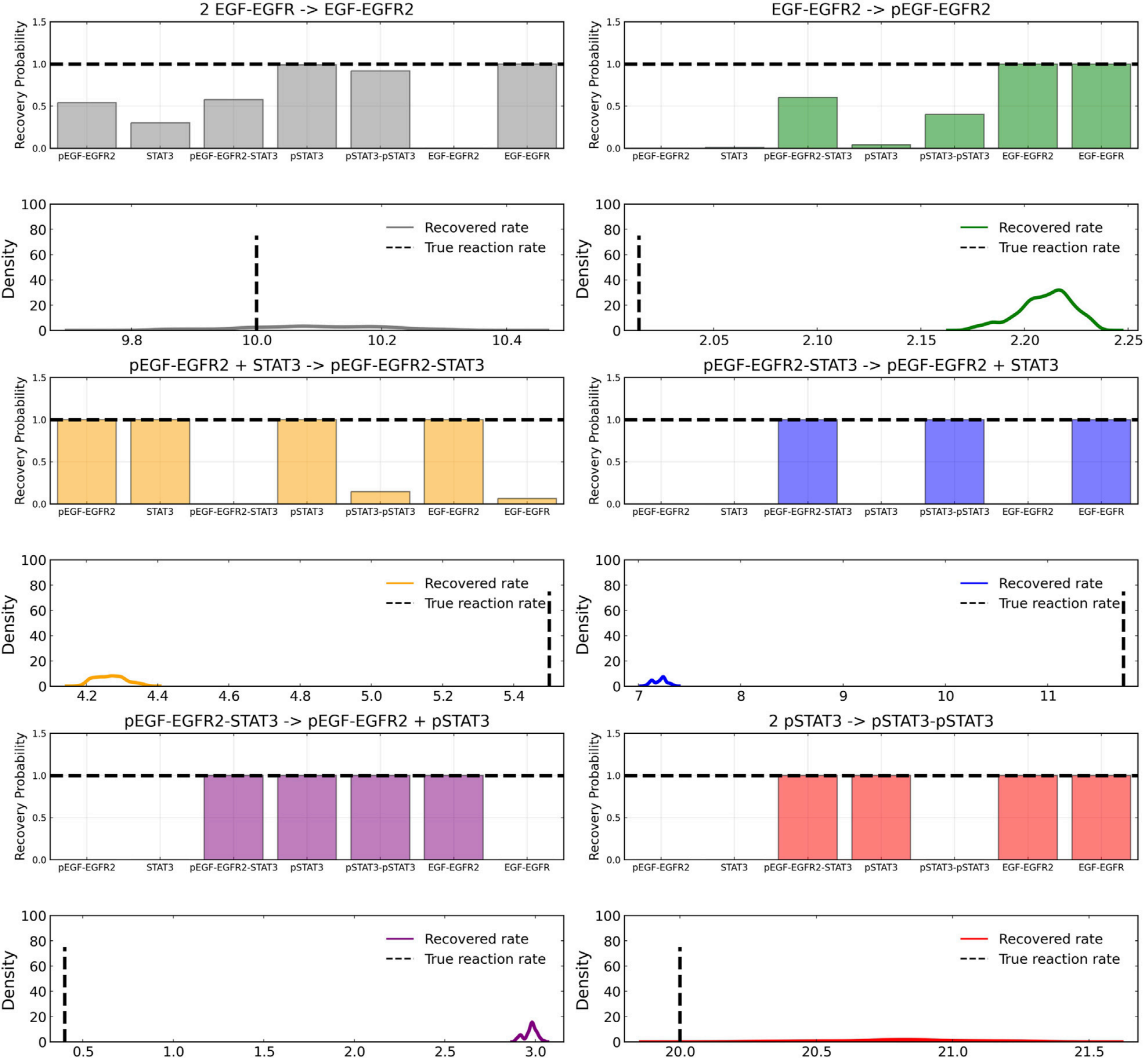
Reactant recovery probability for each of the seven species included in case 2 and posterior distributions of the learned reaction rates for each of the case 2 reactions. Gaussian noise with standard deviation of 5% was added to the training data. A posterior set of 1,000 samples was chosen for the estimation.

After training, the Bayesian CRNN accurately captures the time course for all species in the simplified EGFR-STAT3 pathway and is robust to noise as shown in Figure 10 and from the score plots in Figure 11, we can see the CRNN also correctly identifies the reactants of all the reactions. Figure 12 shows the uncorrected reactant probabilities and the probability densities for the reaction rates. The true reaction rates are contained within the probability density functions of reactions 1 and 6 depicted in Figure 12 a and f, but the other reactions do not include the true rates despite the time courses of the reactants and products being accurately predicted, demonstrating a potential identifiability issue. However, the average percent deviation from the true rates across a posterior set of 1,000 samples remains low, below 40%, for all reactions except reaction 5 which overestimated the rate six-fold, as shown in Table 3. Reactions 1, 2, and 6 did particularly well with an average percent deviation of 1.22% ± 0.81, 9.74% ± 0.64, and 4.08% ± 1.28 respectively.

**TABLE 3:** Average percent deviations from true reaction rates for case 2.

Equation	Average percent deviation
*EGF* − *EGFR* + *EGF* − *EGFR* → *EGF* − *EGFR*2	1.22% ± 0.81
*EGF* − *EGFR*2 → *pEGF* − *EGFR*2	9.74% ± 0.64
*pEGF* − *EGFR*2 + *STAT*3 → *pEGF* − *EGFR*2 − *STAT*3	22.41% ± 0.77
*pEGF* − *EGFR*2 − *STAT*3 → *pEGF* − *EGFR*2 + *STAT*3	38.59% ± 0.56
*pEGF* − *EGFR*2 − *STAT*3 → *pEGF* − *EGFR*2 + *pSTAT*3	643.32% ± 8.92
*pSTAT*3 + *pSTAT*3 → *pSTAT*3 − *pSTAT*3	4.08% ± 1.28

## 4 Discussion

In this study, we combine the previously published chemical reaction neural network (CRNN) Ji and Deng (2021) with a preconditioned Stochastic Gradient Langevin Descent (pSGLD) Markov Chain Monte Carlo (MCMC) stepper Li et al. (2015) of neural ODEs, allowing for the efficient discovery of chemical reaction networks from concentration time course data and quantification of the uncertainty in learned parameters. The specialized form of the CRNN is constrained to satisfy the kinetic equations of reactions, which enables the identification of the learned neural network parameters as the stoichiometric coefficients and reaction rates of the reactions while the Bayesian optimizer provides an estimate of the uncertainty of those parameters.

We tested the algorithm with a simple system of four reactions and five species. With no noise added to the training data, the algorithm perfectly captured the reaction network and accurately matched the time course for each species. Additionally, the posterior distributions of the reaction rates were centered around the true reaction rates. With the addition of noise (standard deviation of 5% of the true concentration) to the training data, the reaction networks were also accurately captured, however the posterior distributions of the reaction rates were no longer centered at the true value but still contained within the distribution. Even with the addition of a large amount of noise (standard deviation of 50%), the reactants of each reaction were correctly identified, and posterior distributions still contained the true reaction rates. To obtain posterior distributions centered at the true value in cases with added noise it is likely that more data is needed as there are various combinations of reaction rates that minimize the loss between the training data and the learned time courses.

We demonstrated that the preconditioned SGLD is necessary to increase training robustness by comparing the results of our Bayesian CRNN trained with the preconditioned SGLD optimizer to a Bayesian CRNN trained with a standard SGLD optimizer and found that not only is the algorithm more efficient and requires less epochs to train but it also more accurately approximates the posterior. The standard SGLD shows high confidence in the incorrect value while the pSGLD in general shows lower confidence but includes the true values.

Additionally, we demonstrated that the Bayesian CRNN in addition to being interpretable, improves upon prediction accuracy when extrapolated beyond the time range used for training by comparing the CRNN to a purely data-driven neural ODE model and an LSTM-based model that contain no prior scientific knowledge about the structure of chemical reactions.

Application of this model to a larger system, a simplified representation of the EGFR-STAT3 pathway containing seven species and six reactions revealed the limitations of this model. While the model was able to accurately predict the time course of all species and learn the correct reactions, as evaluated by correct prediction of reactants and products, the posterior distributions of the reaction rates for four out of six reactions did not include the true rates. This is not completely unexpected since as reaction networks become larger, different combinations of reaction parameters may result in the same concentration dynamics. Future work will be needed to solve this identifiability issue, allowing for the use of this model on larger reaction systems.

The combination of a preconditioned SGLD optimizer with the CRNN allows for efficient training and posterior sampling as well as reliable estimates of parametric uncertainty while accounting for epistemic uncertainty in a way that builds confidence in the learned reaction network and can help determine if more data is needed. This work demonstrates that knowledge-embedded machine learning techniques via SciML approaches may greatly outperform purely deep learning methods in a small-medium data regime that is common in Quantitative Systems Pharmacology (QSP) and demonstrates viable techniques for the automated discovery of QSP models directly from timeseries data.

## Data Availability

Publicly available datasets were analyzed in this study. This data can be found here: https://github.com/nievesemily8/Bayesian_CRNN.
