# Supplementary material for: Uncertainty quantified discovery of chemical reaction systems via Bayesian scientific machine learning
Source: Front Syst Biol. 2024 Mar 8;4:1338518. doi: 10.3389/fsysb.2024.1338518 (PMC12341973; doi:10.3389/fsysb.2024.1338518)
Supplement: Supplementary file 1 [file DataSheet1.PDF]

frontiers

Supplementary Material

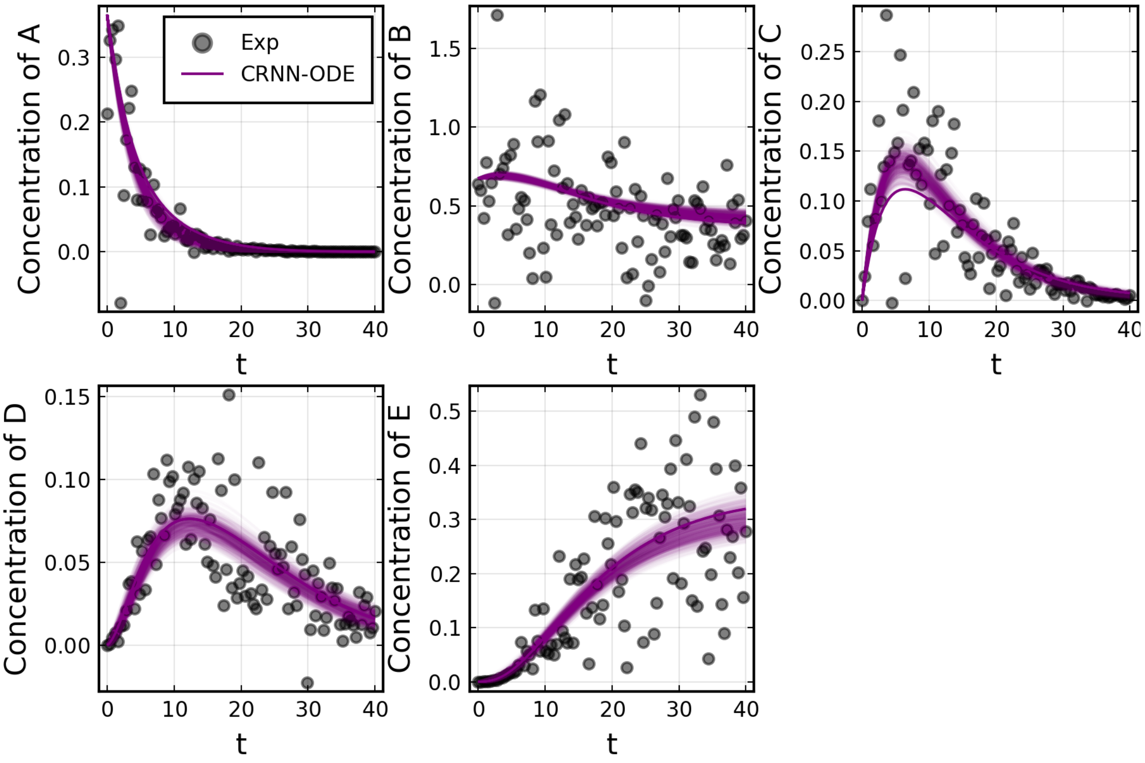

Figure S1. Comparison of the Bayesian chemical reaction neural network prediction compared to the data for the species A, B, C, D, and E in the four reactions described in Case 1. The model was trained with highly noisy data. The standard deviation of the noise is set to 50% of the concentrations. A total of 500 posterior sample predictions are superimposed on the data.

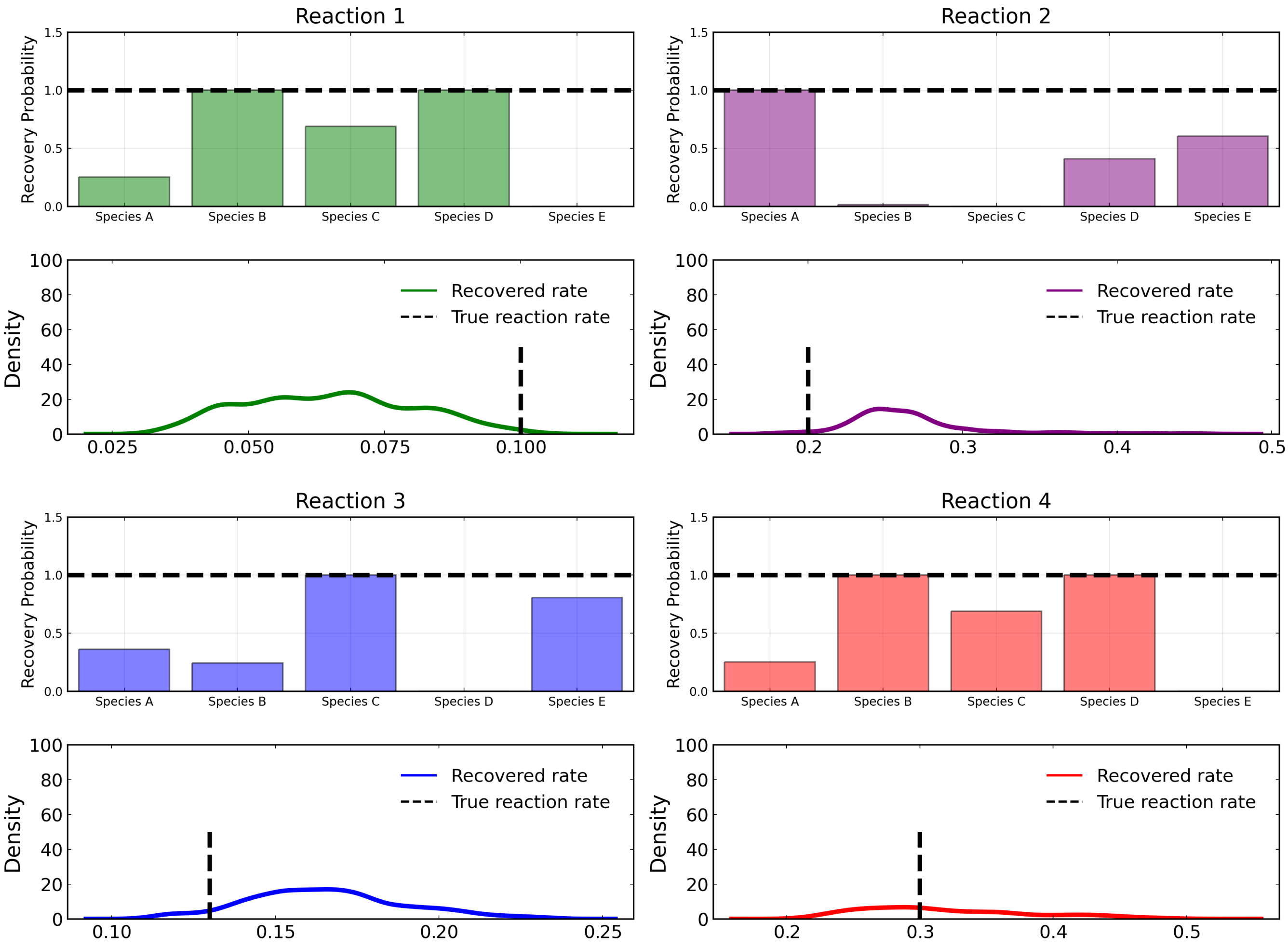

Figure S2. Reactant recovery probability of species A, B, C, D, and E and posterior distributions of learned reaction rates for the four reactions described in Case 1. The model was trained with highly noisy data. The standard deviation of the noise is set to 50% of the concentrations. A posterior set of 1000 samples was chosen for the estimation.

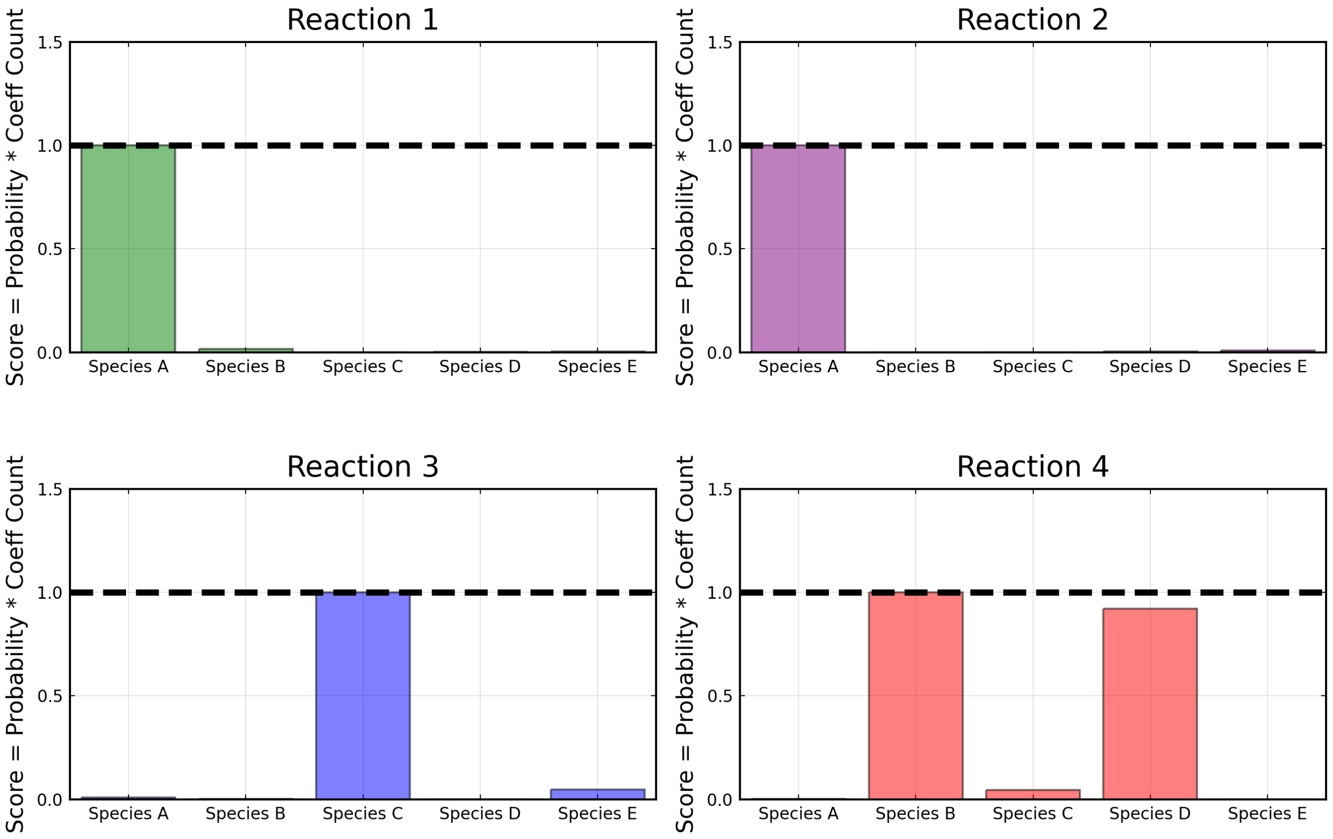

Figure S3. Recovered score metric for each of the four reactions shown in case 1. Score metrics are calculated using equation 6 and represent the weighted probability that a species is a reactant of each reaction. The model is trained with highly noisy data, where the standard deviation of the noise is set to 50% of the concentrations.

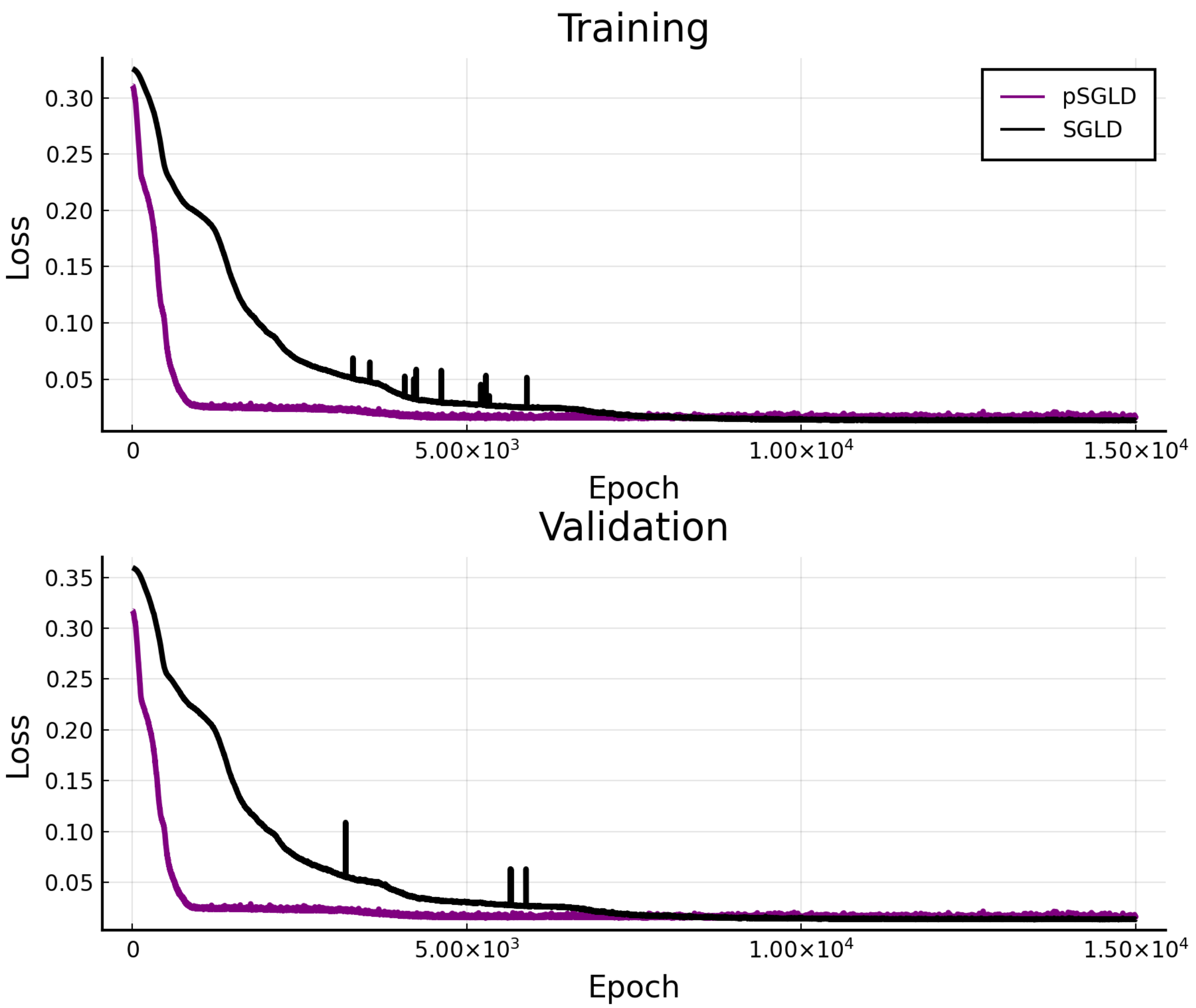

Figure S4. Comparison of training and validation losses over epochs with the preconditioned SGLD optimizer and SGLD optimizer for case 1 with 5% noise added.

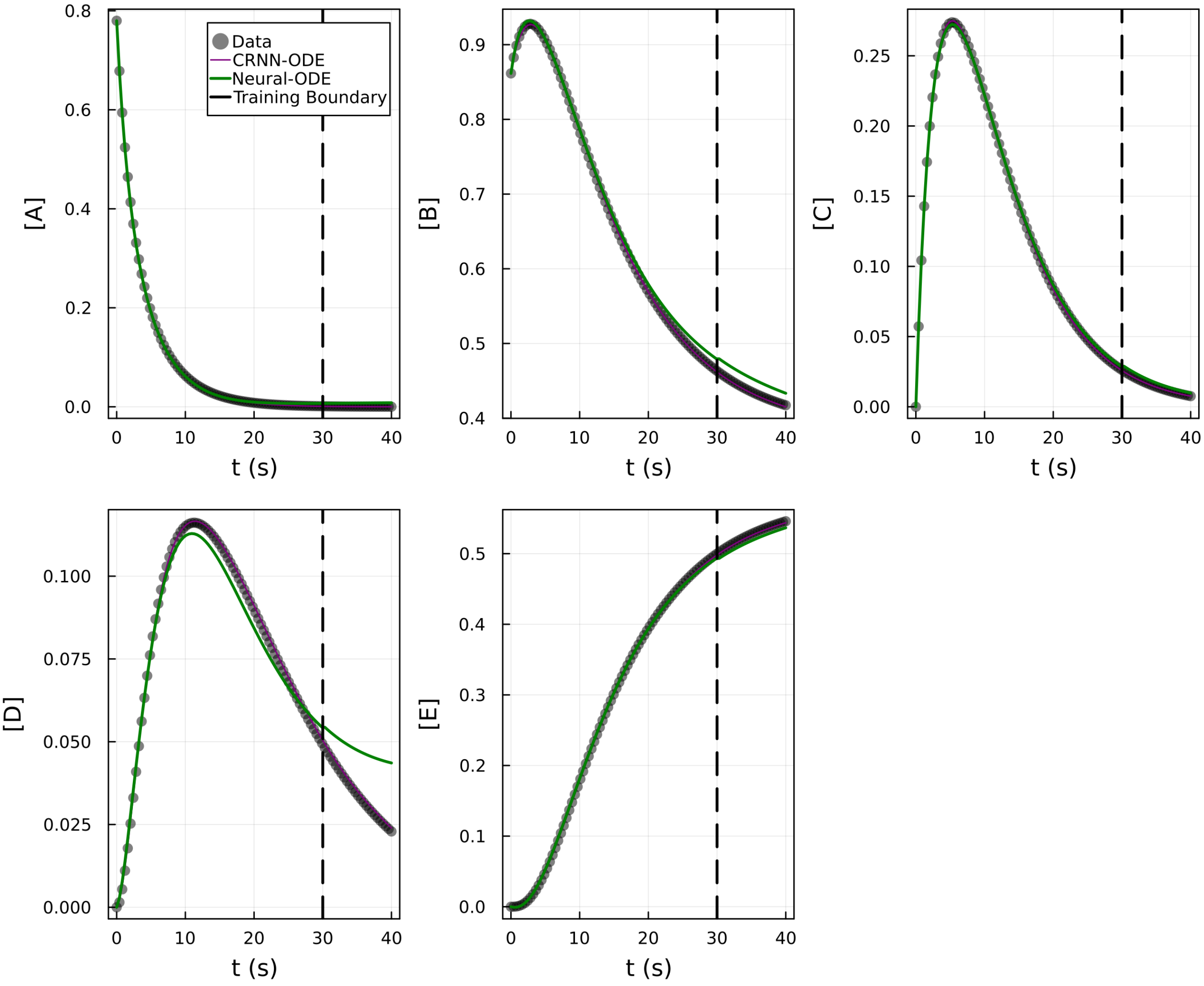

Figure S5. Comparison of Neural ODE with Bayesian CRNN (chemical reaction neural network) Only time points 0-30 were used for training as depicted by the training boundary. Time points 30-40 were used for comparison of the accuracy of extrapolation beyond training time points.
